# Loss of eIF4E Phosphorylation Engenders Depression-like Behaviors via Selective mRNA Translation

**DOI:** 10.1523/JNEUROSCI.2673-17.2018

**Published:** 2018-02-21

**Authors:** Inês S. Amorim, Sonal Kedia, Stella Kouloulia, Konstanze Simbriger, Ilse Gantois, Seyed Mehdi Jafarnejad, Yupeng Li, Agniete Kampaite, Tine Pooters, Nicola Romanò, Christos G. Gkogkas

**Affiliations:** ^1^Centre for Discovery Brain Sciences, University of Edinburgh, Edinburgh EH8 9XD, United Kingdom,; ^2^Patrick Wild Centre, University of Edinburgh, Edinburgh EH8 9XD, United Kingdom,; ^3^Goodman Cancer Research Centre and Biochemistry Department, McGill University, Montréal, Quebec H3A 1A3, Canada, and; ^4^Simons Initiative for the Developing Brain, University of Edinburgh, Edinburgh EH8 9XD, United Kingdom

**Keywords:** depression, eIF4E, inflammation, phospho-eIF4E, translation

## Abstract

The MAPK/ERK (mitogen-activated protein kinases/extracellular signal-regulated kinase) pathway is a cardinal regulator of synaptic plasticity, learning, and memory in the hippocampus. One of major endpoints of this signaling cascade is the 5′ mRNA cap binding protein eIF4E (eukaryotic Initiation Factor 4E), which is phosphorylated on Ser 209 by MNK (MAPK-interacting protein kinases) and controls mRNA translation. The precise role of phospho-eIF4E in the brain is yet to be determined. Herein, we demonstrate that ablation of eIF4E phosphorylation in male mice (*4Eki* mice) does not impair long-term spatial or contextual fear memory, or the late phase of LTP. Using unbiased translational profiling in mouse brain, we show that phospho-eIF4E differentially regulates the translation of a subset of mRNAs linked to inflammation, the extracellular matrix, pituitary hormones, and the serotonin pathway. Consequently, *4Eki* male mice display exaggerated inflammatory responses and reduced levels of serotonin, concomitant with depression and anxiety-like behaviors. Remarkably, eIF4E phosphorylation is required for the chronic antidepressant action of the selective serotonin reuptake inhibitor fluoxetine. Finally, we propose a novel phospho-eIF4E-dependent translational control mechanism in the brain, via the GAIT complex (gamma IFN activated inhibitor of translation). In summary, our work proposes a novel translational control mechanism involved in the regulation of inflammation and depression, which could be exploited to design novel therapeutics.

**SIGNIFICANCE STATEMENT** We demonstrate that downstream of the MAPK (mitogen-activated protein kinase) pathway, eukaryotic Initiation Factor 4E (eIF4E) Ser209 phosphorylation is not required for classical forms of hippocampal LTP and memory. We reveal a novel role for eIF4E phosphorylation in inflammatory responses and depression-like behaviors. eIF4E phosphorylation is required for the chronic action of antidepressants, such as fluoxetine in mice. These phenotypes are accompanied by selective translation of extracellular matrix, pituitary hormones, and serotonin pathway genes, in eIF4E phospho-mutant mice. We also describe a previously unidentified translational control mechanism in the brain, whereby eIF4E phosphorylation is required for inhibiting the translation of gamma IFN activated inhibitor of translation element-containing mRNAs. These findings can be used to design novel therapeutics for depression.

## Introduction

MAPK/ERK (mitogen-activated protein kinases/extracellular signal-regulated kinases) is a conserved signaling pathway, which in response to a plethora of intracellular and extracellular signals, such as cytokines, mitogens, growth factors, hormones, and neurotransmitters, elicits changes in cellular gene-expression programs (Kelleher et al., 2004; Thomas and Huganir, 2004). In the brain, activation of MAPK/ERK in response to excitatory glutamatergic signaling has been linked to regulation of synaptic plasticity, learning, and memory (English and Sweatt, 1997; Zhu et al., 2002; Kelleher et al., 2004; Thomas and Huganir, 2004). Indeed, LTP of excitatory synaptic transmission, mainly in the mammalian hippocampus, requires MAPK/ERK activity (Kanterewicz et al., 2000; Kelleher et al., 2004). Accordingly, MAPK/ERK inhibition impairs learning and hippocampal spatial memory (Atkins et al., 1998) and fear conditioning in rodents (Schafe et al., 2000).

Downstream of MAPK/ERK, the MNK1/2 kinases regulate mRNA translation (Joshi and Platanias, 2014) mainly by phosphorylating eIF4E on Ser209 (Flynn and Proud, 1995; Joshi et al., 1995). eIF4E binds to the mRNA 5′ cap, and together with eIF4G (scaffolding protein) and eIF4A (mRNA helicase), form the eIF4F complex, promoting translation initiation (Hinnebusch et al., 2016). eIF4E stimulates the translation of a subset of mRNAs (“eIF4E-sensitive”), without upregulating global protein synthesis (Hinnebusch et al., 2016). eIF4E, apart from its primary cap binding function, also promotes mRNA restructuring and initiation by stimulating eIF4A helicase activity (Feoktistova et al., 2013). Thus, eIF4E-sensitive mRNAs contain long and highly structured 5′-untranslated regions (UTRs, e.g., proto-oncogenes and growth factors), which require elevated helicase activity for their translation (Sonenberg and Hinnebusch, 2009).

Most of the current literature posits that eIF4E phosphorylation promotes mRNA translation (Pyronnet et al., 1999; Lachance et al., 2002; Panja et al., 2014; Bramham et al., 2016). It was also suggested that eIF4E phosphorylation is not required for translation (McKendrick et al., 2001) or that it decreases cap-dependent translation (Knauf et al., 2001). Several studies identified phospho-eIF4E-sensitive mRNAs in cancer models (Furic et al., 2010; Konicek et al., 2011; Robichaud et al., 2015); however, in brain, only a small subset was revealed. In the hippocampus, phospho-eIF4E regulates the translation of *Mmp9* (Gkogkas et al., 2014; Gantois et al., 2017), while in the suprachiasmatic nucleus, phospho-eIF4E controls the translation of *Per1/2* mRNAs (Cao et al., 2015). Interestingly, phospho-eIF4E is a master regulator of Type I IFN production, and thus of the antiviral response, by controlling the translation of *NFKBIA* mRNA (coding for IκBα protein; nuclear factor of κ light polypeptide gene enhancer in B-cells inhibitor, α) (Herdy et al., 2012). Ablation of phospho-eIF4E downregulates IκBα and activates the transcription factor NF-κB, which regulates cytokine production and antiviral responses (Herdy et al., 2012). Whereas phospho-eIF4E has been implicated in the regulation of some brain functions (Gkogkas et al., 2014; Cao et al., 2015), its precise role in the brain is yet to be elucidated. Little is also known about the subset of phospho-eIF4E-dependent mRNAs or about the regulatory mechanisms governing their translation in the brain.

Herein, we show that, in mice lacking eIF4E phosphorylation (*4Eki*), hippocampal long-term spatial and fear memory formation, and late LTP (L-LTP) are intact. Using unbiased ribosome profiling in *4Eki* brains, we identified reduced translation of mRNAs coding for extracellular matrix (ECM) proteins and pituitary hormones and, unexpectedly, increased translation of serotonin pathway and ribosomal protein mRNAs. This altered translational landscape in *4Eki* brain is accompanied by exaggerated inflammatory responses and reduced brain serotonin levels. Subsequently, we show that *4Eki* mice display depression-like behaviors, which are resistant to chronic treatment with the selective serotonin reuptake inhibitor (SSRI) antidepressant fluoxetine. We demonstrate a potential mechanism for phospho-eIF4E translational control in the brain, which is mediated by altered gamma IFN activated inhibitor of translation (GAIT)-dependent translation and reduced binding of eIF4A1 to the 5′mRNA cap. Together, these data establish a previously unidentified role for eIF4E phosphorylation in depression due to selective translation of a subset of mRNAs.

## Materials and Methods

### 

#### 

##### Transgenic mice.

All procedures were in accordance with United Kingdom Home Office and Canadian Council on Animal Care regulations and were approved by the University of Edinburgh and McGill University. *eIF4E^Ser209Ala^* mice were previously described (Gkogkas et al., 2014) and were maintained on the C57BL/6J background (backcrossed for >10 generations). For most experiments, male mice 8–12 weeks of age were used (for slice electrophysiology 6- to 8-week-old males were used). Food and water were provided *ad libitum*, and mice were kept on a 12 h light/dark cycle. Pups were kept with their dams until weaning at postnatal day 21. After weaning, mice were group housed (maximum of 4 per cage) by sex. Cages were maintained in ventilated racks in temperature (20°C-21°C) and humidity (∼55%) controlled rooms, on a 12 h circadian cycle (7:00 AM to 7:00 PM light period). For all behavioral testing, mice were handled/habituated for 3–4 consecutive days before experimental testing. Fluoxetine hydrochloride (Sigma-Aldrich) or vehicle (saline) was injected at 10 mg/kg intraperitoneally for 21 d. Lipopolysaccharide (LPS, strain O111:B4; Sigma-Aldrich) or vehicle (saline) was injected at 5 mg/kg intraperitoneally and brains were collected 4 h later.

##### Morris Water Maze (MWM).

Mice were handled for 3 d before the experiment. Training in the pool (100-cm-diameter and 10-cm-diameter platform; water temperature was 24°C) consisted of three trials per day (20 min intertrial interval), where each mouse swam until it reached the hidden platform. Animals that did not find the platform after 60 s were gently guided to it and would remain there for 10 s before returning them to the cage. For the probe test, the platform was removed and animals could swim for 60 s. The swimming trajectory and velocity were monitored with a video tracking system (HVS Image).

##### Contextual fear conditioning (CFC).

Mice were handled for 3–4 d before the start of the experiment and then conditioned in the chamber: 2 min acclimatization to the context, followed by the unconditioned stimulus; one foot shock (0.5 mA, 4 s) followed by a 30 s interval, terminating with another identical foot shock. The mice remained in the chamber for an additional 1 min after the end of the last unconditioned stimulus, after which they were returned to their home cages. Contextual fear memory was assayed 24 h after training by reexposing the animals to the conditioning context for a 5 min period. During this period, the incidence of freezing (immobile except for respiration) was recorded (FreezeFrame, Coulbourn Instruments). Freezing behavior was analyzed by assigning at 5 s intervals as either freezing or not freezing. Data are expressed as the percentage of 5 s intervals scored as “freezing.”

##### Forced Swim Test (FST).

Transparent glass cylinders (50 cm height × 20 cm diameter) were filled with tap water maintained at 25°C. The water depth was adjusted according to the size of the mouse, so that it could not touch the bottom of the container with its hind legs. Animals were tested for 6 min, while only the last 4 min were scored for immobility using a manual timer.

##### Tail Suspension Test (TST).

Each mouse was suspended within its own three-walled (white) rectangular compartment (55 cm height × 15 cm width × 11.5 cm depth) in the middle of an aluminum suspension bar using adhesive tape. The width and depth are sufficiently sized so that the mouse cannot touch the walls. The duration of the test is 5 min and immobility was manually scored with a timer.

##### Novelty Suppressed Feeding (NSF).

Mice were handled for 3 d and following 24 h food deprivation were placed in a 40 × 40 cm^2^ open field arena for 5 min. Weight loss was <7%, and no difference was seen between genotypes. At the center of the arena, two food pellets were fixed on a Whatman paper covered circular platform (replaced between subjects) glued on a 10 m Petri dish, to stop mice from removing the pellets. Animals that did not consume the pellets within the testing period were assigned to a latency of 300 s. The latency to grab food with both limbs and commence eating was measured with a stopwatch, and animals were weighed before the start of the experiment.

##### Open Field Test (OF).

Mice were handled for 3–4 d and then allowed to freely explore a 40 × 40 cm^2^ open field arena for 10 min. A 20 × 20 cm center region was designated as the center square. Time in the center square and outside as well as total distance traveled were recorded.

##### Elevated Plus Maze (EPM).

Mice were handled for 3–4 d and then allowed to freely explore an EPM (50 cm from ground), with open (2) and closed (2) arms: 50 cm length × 10 cm width and 40 cm height for the walls of closed arms. Time spent in the closed and open arms over a period of 5 min was manually recorded using a handheld timer.

##### Extracellular field recordings.

Transverse hippocampal slices (400 μm) were prepared from wild-type (WT) or *4Eki* males (6–8 weeks old). Slices were then allowed to recover submerged for at least 1 h at 32°C in oxygenated ACSF containing 124 mm NaCl, 2.5 mm KCl, 1.25 mm NaH_2_PO_4_, 25 mm NaHCO_3_, 20 mm glucose, 1 mm MgCl_2_, and 2 mm CaCl_2_ before transferring to a recording chamber at 29°C–31°C, which was continuously perfused with ACSF. fEPSPs were recorded in CA1 stratum radiatum with glass electrodes (2–3 mΩ) filled with ACSF. Schaffer collateral fEPSPs were evoked with a twisted bipolar stimulating electrode placed in stratum radiatum proximal to CA3 region. All signals collected were analyzed using WinLTP program. Test pulses were adjusted to obtain 40%–50% maximal fEPSP, delivered every 30 s, and averaged over 1 min. Basal responses were measured 60 min before the LTP stimulus. For the induction of L-LTP, four 1 s trains of 100 Hz high-frequency stimulation were delivered with an intertrain interval of 5 min. The initial slopes of the fEPSPs were measured, and values were normalized to the averaged baseline slope value for each slice. Percentage of potentiation was calculated as the difference between averaged values for a 10 min period before the tetanus and the last 10 min of recording.

##### Immunoblotting.

Dissected brain tissue was homogenized in buffer B (50 mm MOPS/KOH, pH 7.4, 100 mm NaCl, 50 mm NaF, 2 mm EDTA, 2 mm EGTA, 1% NP-40, 7 mm β-mercaptoethanol) supplemented with protease and phosphatase inhibitors (Roche). Samples were incubated on ice for 15 min, with occasional vortexing, and cleared by centrifugation for 20 min at 16,000 × *g* at 4°C. The supernatant was used for Western blotting after the protein concentration of each sample was determined by measuring A280 absorbance on a NanoDrop (ThermoFisher Scientific). The 50 μg of protein per lane was prepared in SDS sample buffer (50 mm Tris, pH 6.8, 100 mm DTT, 2% SDS, 10% glycerol, 0.1% bromophenol blue), heated to 98°C for 5 min, and resolved on 10%–16% polyacrylamide gels. Proteins were transferred to 0.2 μm nitrocellulose membranes (Bio-Rad), blocked in 5% BSA in TBS-T (10 mm Tris, pH 7.6, 150 mm NaCl, 0.1% Tween 20) for 1 h at room temperature, incubated with primary antibodies overnight at 4°C and with secondary antibodies for 1 h at room temperature. Primary antibodies were diluted in 1% BSA in TBS-T containing 0.02% Na azide, and between incubations membranes were washed extensively in TBS-T. Blots were imaged using an Odyssey Imaging System (Li-COR Biosciences) at a resolution of 169 μm and quantified using the ImageStudio Software (Li-COR Biosciences). For quantitative Western blotting, the intensity of each protein band was measured in triplicate to minimize measuring variability. β-actin was used as a loading control. Data are shown as arbitrary units after normalization to control.

##### Antibodies.

The antibodies used for immunoblotting or immunofluorescence are summarized in Table 1.

**Table 1.** Details of antibodies used

Protein	Host species	Supplier	Catalog #	Predicted kDa	WB or IF
β-actin	Mouse	Sigma-Aldrich	A5316	42	1:5000 WB
eIF4A1	Rabbit	Abcam	ab31217	48	1:1000 WB
eIF4E	Mouse	Santa Cruz Biotechnology	sc-271480	29	1:1000 WB, 1:500 IF
eIF4E phospho Ser209	Rabbit	Abcam	ab76256	25	1:500 IF
eIF4G1	Rabbit	Cell Signaling Technology	2498	220	1:1000 WB
RPL13A	Rabbit	Cell Signaling Technology	2765	23	1:500 WB
EPRS	Rabbit	Abcam	ab31531	163	1:1000 WB
GAPDH	Rabbit	Cell Signaling Technology	2118	37	1:1000 WB

##### Immunofluorescence and confocal imaging.

Mice were anesthetized and transcardially perfused with 4% PFA (Electron Microscopy Sciences) in PBS. The brain was immediately dissected from the skull, postfixed in 4% PFA in PBS overnight at 4°C, and cryopreserved in a solution of 30% sucrose in PBS for 48 h at 4°C. Each brain was embedded in a mixture (1:1) of OCT:30% sucrose, and 30 μm coronal sections were cut on a cryostat (Leica). Sections were stored at 4°C as floating sections in PBS with 0.02% Na azide, until used. Sections were then incubated in blocking solution (5% NGS; Cell Signaling Technology), 0.3% Triton X-100 (Sigma-Aldrich) in PBS for 1 h at room temperature, washed briefly in PBS, and incubated with primary antibodies overnight at 4°C and with secondary antibodies for 2 h at room temperature. The antibodies were diluted in 2% NGS, 0.1% Triton X-100 in PBS, and the sections were washed extensively in PBS between incubations. A nuclear counterstain was applied by incubating the sections for 5 min with DAPI solution (1 μg/ml; ThermoFisher Scientific). Sections were mounted on glass slides using PermaFluor Mounting Media (ThermoFisher Scientific), protected with a glass coverslip, and stored at 4°C in the dark. Images were collected on a Zeiss LSM800 confocal microscope.

##### Quantitative ELISA for cytokines and serotonin.

Forebrain tissue was homogenized in kit sample buffer (QiaMouse Inflammatory Cytokines, Generon Iba-1 or Chemokines Multi-Analyte ELISArray Kit, QIAGEN and Serotonin ELISA kit, Enzo Life Sciences) with ∼30 strokes in a glass Dounce homogenizer on ice. Lysates were centrifuged at 16,000 × *g* for 5 min, and the supernatant was used for the assay. Detection was performed as per each kit's guidelines. For both assays, 50 μg of total protein was analyzed per sample (measured by Bradford assay, Bio-Rad). Optical density values were converted to pg/mg of total protein using curves of OD versus kit standard cytokine concentrations. In brain tissue, we detected the following cytokines from the kit: IL1B, IL2, IL6, IL10, IFNγ and TNFα, and ionized calcium-binding adapter molecule-1 (Iba-1) and serotonin as pg/mg of tissue.

##### Ribosome profiling and bioinformatics analysis.

Flash-frozen forebrain tissue was pulverized using liquid nitrogen and then lysed in hypotonic buffer: 5 mm Tris-HCl, pH 7.5, 2.5 mm MgCl_2_, 1.5 mm KCl, 1× protease inhibitor mixture (EDTA-free), 100 μg/ml cycloheximide (Sigma-Aldrich), 2 mm DTT 0.5% (w/v) Triton X-100, and 0.5% (w/v) sodium deoxycholate, to isolate the polysomes with centrifugation (20,000 × *g*) at 4°C for 5 min. Ribosome profiling was performed as previously described (Ingolia et al., 2012), with minor modifications. Briefly, 500 μg of the lysed RNPs (forebrain tissue) was subjected to ribosome footprinting by RNase I treatment at 4°C for 45 min with gentle mixing. Monosomes were pelleted by ultracentrifugation in a 34% sucrose cushion at 70,000 RPM for 3 h, and RNA fragments were extracted twice with acid phenol, once with chloroform, and precipitated with isopropanol in the presence of NaOAc and GlycoBlue. Purified RNA was resolved on a denaturing 15% polyacrylamide urea gel, and the section corresponding to 28–32 nucleotides containing the ribosome footprints was excised, eluted, and precipitated by isopropanol; 100 μg of cytoplasmic RNA was used for mRNA-Seq analysis. Poly(A)^+^ mRNAs were purified using magnetic oligo-dT DynaBeads (Invitrogen) according to the manufacturer's instructions. Purified RNA was eluted from the beads and mixed with an equal volume of 2× alkaline fragmentation solution (2 mm EDTA, 10 mm Na_2_CO_3_, 90 mm NaHCO_3_, pH 9.2) and incubated for 20 min at 95°C. Fragmentation reactions were mixed with stop/precipitation solution (300 mm NaOAc, pH 5.5, and GlycoBlue), followed by isopropanol precipitation. Fragmented mRNA was size-selected on a denaturing 10% polyacrylamide urea gel, and the area corresponding to 35–50 nucleotides was excised, eluted, and precipitated with isopropanol. All samples were analyzed on a Bioanalyzer High Sensitivity DNA chip (Agilent Technologies) to confirm expected size range and quantity and sequenced on a HiSeq 2500 system (Illumina). Raw sequencing data were demultiplexed by the sequencing facility (Genome Quebec). Sequences were analyzed using a custom-developed bioinformatics pipeline adapted from Ingolia et al. (2012). Reads were adapter-trimmed using the FASTX toolkit, contaminant sequences (rRNA, tRNA) removed using bowtie, and reads aligned to a reference genome using STAR. Cufflinks was used to quantify reads and calculate reads per kilobase of transcript per million mapped reads (RPKM) for each transcript. Translational efficiency (TE) for each transcript was calculated by dividing RPKM values of the RPF libraries by RPKM values of the total RNA libraries. Changes in TE and transcription (mRNA RPKM) values were analyzed for predefined pairwise comparisons between experimental groups (for review, see Quackenbush, 2002). First, averages were calculated for replicate TE/RPKM values of each treatment on a per-gene basis using the geometric mean. From these averages, two statistics were derived for each gene: (1) ratio, defined as the quotient of values for alternative treatment (e.g., knock-in) and base level treatment (e.g., WT); and (2) intensity, defined as the product of the aforementioned values. Data were ordered by increasing log_10_(Intensity). Along this ordered set of values, mean log_10_ (Intensity) as well as mean and SD of log_2_(Ratio) were calculated within a sliding window of 100 genes at steps of 50 genes. Each gene was assigned to the window with a mean log_10_ (Intensity) closest to the gene's log_10_(Intensity). A *z* score was then calculated for the *i*^th^ gene using its window's log_2_(Ratio) mean and SD as follows:

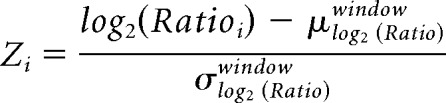
 A *p* value was derived from the *z* score of the *i*^th^ gene by treating the latter as a quantile of the standard normal distribution as follows:

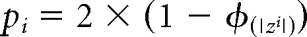
 False-discovery rates (FDRs) were calculated from *p* values derived with the *z* score as in Reiner et al. (2003). Genes with <128 reads were discarded. Raw RNAseq data will be deposited to NCBI Gene Expression Omnibus.

##### Principal components analysis (PCA) and hierarchical clustering.

PCA was conducted with R package vegan version 2.4.4. Genes with undefined log2-transformed values (for RPKM = 0 or TE = 0) were excluded from the analysis. log_2_-transformed values of the remaining set of genes were standardized on a per-gene basis (scaled to mean = 0 and SD = 1). Euclidean distances of samples (replicates) were calculated from the same standardized log2-transformed gene data used in PCA. Hierarchical clustering based on the complete-linkage algorithm was performed on the distance matrix with R package stats version 3.4.2. Results were visualized as dendrograms below the corresponding PCA plot.

##### g:profiler analysis of mRNAs.

Functional enrichment analysis was performed using the g:Ghost package of g:profiler to assign gene ontology categories to ribosome profiling lists of differentially translated genes (Reimand et al., 2016). Hierarchical filtering was used: best per parent group-strong. The probability threshold for all functional categories was set at 0.05, using correction for multiple testing with the g:SCS algorithm (Reimand et al., 2016).

##### UTR sequence analysis.

UTR sequence analysis was performed using RegRNA (Huang et al., 2006). Motifs in 5′- and 3′-UTR were detected with default parameters; 652 downregulated, 52 upregulated, and 325 control mRNA UTRs were obtained from Biomart ENSEMBL (Yates et al., 2016) using the GRCm38.p5 version of the mouse genome. Length in BP and %GC content were calculated using free Python-based scripts (Multifastats; https://github.com/davidrequena/multifastats).

##### Cap column pulldown.

Forebrain tissue was dissected and lysates were prepared in the same way as for immunoblotting (see above); 500 μg of protein was incubated with 50 μl of m^7^GDP agarose (Jena Biosciences), in a total volume of 1 ml buffer C (50 mm MOPS-KOH, pH 7.4, 100 mm NaCl, 50 mm NaF, 0.5 mm EDTA, 0.5 mm EGTA, 7 mm β-mercaptoethanol, 0.5 mm PMSF, 1 mm Na_3_VO_4_, and 0.1 mm GTP), for 90 min at 4°C with rotation. The beads were washed three times in buffer C, and the cap-bound fraction was eluted in 50 μl of 2× SDS sample buffer with boiling at 70°C for 10 min.

##### Experimental design and statistical analysis.

Experimenters were blinded to the genotype during testing and scoring. All data are presented as mean ± SEM (error bars), and individual experimental points are depicted in column or bar graphs. Statistical significance was set a priori at 0.05. Fluoxetine treatment was randomized across cages (not all animals in one cage received the same treatment; vehicle or fluoxetine). No nested data were obtained in this study; we only collected one observation per research object. Details for statistical tests used were provided within figure legends or the relative methods description and summarized in Table 2. Data summaries and statistical analysis were performed using GraphPad Prism 6 and SPSS version 21 (IBM).

**Table 2.** Statistical analysis

Test	Mean ± SEM	Significance and multiple comparisons	Parameter	*N*	Descriptive statistics	Figure
Repeated-measures ANOVA, with Tukey's post hoc	WT: 28.404 ± 1.700	Day: *p* < 0.001	Latency (s)	WT (7), 4Eki (8)	Day: *F*_(4,68)_ = 39.900	1*B*
	4Eki: 27.630 ± 1.792	Genotype: *p* = 0.758			Genotype: *F*_(1,17)_ = 0.098	
		Day × Genotype: *p* = 0.668			Day × Genotype: *F*_(4,68)_ = 0.595	
	Target quadrant:	Quadrant: *p* < 0.001	No. of platform crossings		Day: *F*_(4,68)_ = 39.900	
	WT: 6.857 ± 0.519	Genotype: *p* = 0.960			Genotype: *F*_(1,17)_ = 0.098	
	4Eki: 6.500 ± 0.486	Quadrant × Genotype: *p* = 0.578			Day × Genotype: *F*_(4,68)_ = 0.595	
	Right quadrant:					
	WT: 3.857 ± 0.519					
	4Eki 3.375 ± 0.486					
	Opposite quadrant:					
	WT: 3.286 ± 0.519					
	4Eki: 3.250 ± 0.486					
	Left quadrant:					
	WT: 3.571 ± 0.519					
	4Eki: 4.375 ± 0.486					
	Target quadrant:	Quadrant: *p* < 0.001	Quadrant occupancy (%)		Quadrant: *F*_(3,52)_ = 18.160	
	WT: 32.286 ± 1.922	Genotype: *p* = 0.946			Genotype: *F*_(1,52)_ = 0.005	
	4Eki: 33.625 ± 1.798	Quadrant × Genotype: *p* = 0.756			Quadrant × Genotype: *F*_(3,52)_ = 0.396	
	Right quadrant:					
	WT: 24.714 ± 1.922					
	4Eki: 23.00 ± 1.798					
	Opposite quadrant:					
	WT: 23.571 ± 1.922					
	4Eki: 22.250 ± 1.798					
	Left quadrant:					
	WT: 19.286 ± 1.922					
	4Eki: 20.625 ± 1.798					
Student's *t* test	WT: 30.75 ± 6.35	*p* = 0.077	Freezing (%)	WT (8), 4Eki (8)	Genotype: *F*_(1,19)_ = 0.088	1*C*
	4Eki: 3.57 ± 7.02					
Student's *t* test	WT: 13.44 ± 4.337	*p* = 0.968	% potentiation	WT (7), 4Eki (6)	*t* = 3.551; df = 13	1*E*
	4Eki: 13.18 ± 4.911					
One-way ANOVA with Tukey's post hoc	Down: 247.0 ± 6.660	Up versus down: *p* = 0.005	5′-UTR length	Down (651), up (52), control (325),	*F*_(3,53702)_ = 7.255C	2*D*
	Up: 138.0 ± 29.790	Control versus down: *p* < 0.001				
	Control: 188.0 ± 14.530	Whole genome versus down: *p* = 0.014				
	Whole genome: 219.7 ± 1.008	Control versus up: *p* = 0.468				
		Whole genome versus up: *p* = 0.0524				
		Whole genome versus Control: *p* = 0.0645				
	Down: 59.38 ± 0.555	Up versus down: *p* = 0.663	5′-UTR GC % content		*F*_(3,53310)_ = 2.018	
	Up: 61.46 ± 1.492	Control versus down: *p* = 0.453				
	Control: 60.85 ± 0.602	Whole genome versus down: *p* > 0.999				
	Whole genome: 59.43 ± 0.052	Control versus up: *p* = 0.986				
		Whole genome versus up: *p* = 0.611				
		Whole genome versus Control: *p* = 0.141				
	Down: 1112 ± 89.52	Up versus down: *p* = 0.998	3′-UTR length		*F*_(3,49949)_ = 2.146	
	Up: 1095 ± 150.6	Control versus down: *p* = 0.452				
	Control: 1293 ± 91.93	Whole genome versus down: *p* = 0.998				
	Whole genome: 1095 ± 6.326	Control versus up: *p* = 0.780				
		Whole genome versus up: *p* > 0.999				
		Whole genome versus Control: *p* = 0.055				
	Down: 46.34 ± 0.534	Up versus down: *p* = 0.7808	3′-UTR GC % content		*F*_(3,49949)_ = 1.963	
	Up: 45.07 ± 0.877	Control versus down: *p* = 0.214				
	Control: 44.86 ± 0.458	Whole genome versus down: *p* = 0.073				
	Whole genome: 44.91 ± 0.039	Control versus up: *p* = 0.998				
		Whole genome versus up: *p* = 0.999				
		Whole genome versus Control: *p* = 0.999				
One-way ANOVA with Bonferroni's post hoc	WTveh-IFNγ: 0.011 ± 0.001	WTveh-IFNγ versus WTlps-IFNγ: *p* = 0.003	Concentration (pg/mg protein)	WT (10), 4Eki (10)	*F*_(3,36)_ = 52.02	3*A*
	WTlps-IFNγ: 0.024 ± 0.002	WTveh-IFNγ versus 4Ekiveh-IFNγ: *p* = 0.122				
	4Ekiveh-IFNγ: 0.019 ± 0.002	WTveh-IFNγ versus 4Ekilps-IFNγ: *p* < 0.001				
	4Ekilps-IFNγ: 0.052 ± 0.003	WTlps-IFNγ versus 4Ekiveh-IFNγ: *p* > 0.999				
		WTlps-IFNγ versus 4Ekilps-IFNγ: *p* < 0.001				
		4Ekiveh-IFNγ versus 4Ekilps-IFNγ: *p* < 0.001				
	WTveh-IL-2: 1.927 ± 0.064	WTveh-IL-2 versus WTlps-IL-2: *p* < 0.001			*F*_(3,36)_ = 266.7	
	WTlps-IL-2: 7.412 ± 0.177	WTveh-IL-2 versus 4Ekiveh-IL-2: *p* < 0.001				
	4Ekiveh-IL-2: 3.344 ± 0.230	WTveh-IL-2 versus 4Ekilps-IL-2: *p* < 0.001				
	4Ekilps-IL-2: 8.908 ± 0.273	WTlps-IL-2 versus 4Ekiveh-IL-2: *p* < 0.001				
		WTlps-IL-2 versus 4Ekilps-IL-2: *p* < 0.001				
		4Ekiveh-IL-2 versus 4Ekilps-IL-2: *p* < 0.001				
	WTveh-TNFα: 2.728 ± 0.289	WTveh-TNFα versus WTlps-TNFα: *p* < 0.001			*F*_(3,36)_ = 208.7	
	WTlps-TNFα: 9.028 ± 0.240	WTveh-TNFα versus 4Ekiveh-TNFα: *p* < 0.001				
	4Ekiveh-TNFα: 7.062 ± 0.283	WTveh-TNFα versus 4Ekilps-TNFα: *p* < 0.001				
	4Ekilps-TNFα: 12.80 ± 0.339	WTlps-TNFα versus 4Ekiveh-TNFα: *p* < 0.001				
		WTlps-TNFα versus 4Ekilps-TNFα: *p* < 0.001				
		4Ekiveh-TNFα versus 4Ekilps-TNFα: *p* < 0.001				
	WTveh-IL-6: 1.422 ± 0.096	WTveh-IL-6 versus WTlps-IL-6: *p* < 0.001			*F*_(3,36)_ = 102.5	
	WTlps-IL-6: 4.467 ± 0.128	WTveh-IL-6 versus 4Ekiveh-IL-6: *p* > 0.999				
	4Ekiveh-IL-6: 1.602 ± 0.126	WTveh-IL-6 versus 4Ekilps-IL-6: *p* < 0.001				
	4Ekilps-IL-6: 4.049 ± 0.240	WTlps-IL-6 versus 4Ekiveh-IL-6: *p* < 0.001				
		WTlps-IL-6 versus 4Ekilps-IL-6: *p* = 0.414				
		4Ekiveh-IL-6 versus 4Ekilps-IL-6: *p* < 0.001				
	WTveh-IL-1β: 11.32 ± 0.290	WTveh-IL-1β versus WTlps-IL-1β: *p* < 0.001			*F*_(3,36)_ = 132.1	
	WTlps-IL-1β: 27.47: 27.47 ± 0.687	WTveh-IL-1β versus 4Ekiveh-IL-1β: *p* > 0.999				
	4Ekiveh-IL-1β: 11.03 ± 0.830	WTveh-IL-1β versus 4Ekilps-IL-1β: *p* < 0.001				
	4Ekilps-IL-1β: 25.24 ± 1.007	WTlps-IL-1β versus 4Ekiveh-IL-1β: *p* < 0.001				
		WTlps-IL-1β versus 4Ekilps-IL-1β: *p* = 0.176				
		4Ekiveh-IL-1β versus 4Ekilps-IL-1β: *p* < 0.001				
	WTveh-IL-10: 0.376 ± 0.016	WTveh-IL-10 versus WTlps-IL-10: *p* < 0.001			*F*_(3,36)_ = 135.9	
	WTlps-IL-10: 1.234 ± 0.050	WTveh-IL-10 versus 4Ekiveh-IL-10: *p* > 0.999				
	4Ekiveh-IL-10: 0.397 ± 0.028	WTveh-IL-10 versus 4Ekilps-IL-10: *p* < 0.001				
	4Ekilps-IL-10: 1.222 ± 0.057	WTlps-IL-10 versus 4Ekiveh-IL-10: *p* < 0.001				
		WTlps-IL-10 versus 4Ekilps-IL-10: *p* = 0.414				
		4Ekiveh-IL-10 versus 4Ekilps-IL-10: *p* < 0.001				
Student's *t* test	WT-serotonin: 558.9 ± 22.96	*p* < 0.001	Concentration (pg/mg protein)	WT (20), 4Eki (20)	*t* = 4.025; df = 38	3*B*
	4Eki-serotonin: 431.9 ± 21.64					
One-way ANOVA with Bonferroni's post hoc	Iba-1: WTveh: 1.141 ± 0.1125, WTlps: 4.214 ± 0.2336, 4Ekiveh: 2.686 ± 0.2241, 4Ekilps: 6.315 ± 0.4868	WTveh versus WTlps: *p* < 0.001	Concentration (pg/mg protein)	WT (10), 4Eki (10)	*F*_(3,36)_ = 55.02	3*C*
		WTveh versus 4Ekiveh: *p* = 0.0047				
		WTveh versus 4Ekilps: *p* < 0.001				
		WTlps versus 4Ekiveh: *p* = 0.0053				
		WTlps versus 4Ekilps: *p* < 0.001				
		4Ekiveh versus 4Ekilps: *p* < 0.001				
Student's *t* test	WT: 83.05 ± 10.00	*p* = 0.015	Immobility (s) FST	WT (19), 4Eki (18)	*t* = 2.548; df = 35	4*A*
	4Eki: 121.06 ± 11.10					
	WT: 150.50 ± 9.91	*p* < 0.001	Immobility (s) TST	WT (14), 4Eki (18)	*t* = 4.761; df = 30	
	4Eki: 213.39 ± 8.74					
	WT: 71.67 ± 15.24	*p* = 0.0015	Latency to consume food (s) NSF	WT (18), 4Eki (18)	*t* = 3.447; df = 34	
	4Eki: 143.6 ± 14.23					
Student's *t* test	WT center: 69.90 ± 5.923	*p* < 0.001, *p* < 0.001	Time spent in center (s), Time spent in proximity of walls or corners (s)	WT (10), 4Eki (12)	*t* = 6.792; df = 20, *t* = 10.32; df = 20	
	4Eki center: 25.00 ± 3.492 WTwall/corner: 126.9 ± 14.93 4Eki wall/corner: 372.7 ± 17.80					
	WT: 403.2 ± 17.04	*p* = 0.801	Time spent outside center (s)		*t* = 0.199; df = 20	4*B*
	4Eki: 396.8 ± 18.27					
	WT: 4193 ± 125.8	*p* = 0.830	Distance travelled (cm)		*t* = 0.216; df = 20	
	4Eki: 4233 ± 133.3					
One-way ANOVA with Bonferroni's post hoc	WT: open 98.63 ± 5.227, closed 110.3 ± 5.876 4Eki: open 32.38 ± 10.97, closed 189.8 ± 5.786	*p* < 0.001	Time spent in arms (s)	WT (8), 4Eki (8)	*F*_(3,28)_ = 76.91	4*C*
One-way ANOVA with Bonferroni's post hoc	WT veh: 100.3 ± 3.546	WT veh versus WT fl: *p* = 0.005	Immobility (s) FST	WT (12), 4Eki (12)	*F*_(3,44)_ = 25.320	5*B*
	WT fl: 69.33 ± 4.761	WT veh versus 4Eki veh: *p* = 0.0079				
	4Eki veh: 129.8 ± 7.229	WT veh versus 4Eki fl: *p* < 0.001				
	4Eki fl: 136.2 ± 7.817	WT fl versus 4Eki veh: *p* < 0.001				
		WT fl versus 4Eki fl: *p* < 0.001				
		4Eki veh versus 4Eki fl: *p* > 0.999				
	WT veh: 135.1 ± 11.06	WT veh versus WTfl: *p* = 0.0081	Immobility (s) TST		*F*_(3,44)_ = 73.621	5*C*
	WT fl: 97.75 ± 6.516	WT veh versus 4Eki veh: *p* < 0.001				
	4Eki veh: 229.5 ± 6.763	WT veh versus 4Eki fl: *p* < 0.001				
	4Eki fl: 236.3 ± 7.334	WT fl versus 4Eki veh: *p* < 0.001				
		WT fl versus 4Eki fl: *p* < 0.001				
		4Eki veh versus 4Eki fl: *p* < 0.001				
Student's *t* test	Input: WT:1.090 ± 0.05874, 4Eki: 1.048 ± 0.05977, cap: WT: 0.9975 ± 0.1723, 4EKI: 0.4875 ± 0.1062	Input: *p* = 0.630, cap: *p* = 0.045	rpL13a	WT (4), 4Eki (4) or, WT (8), 4EKi (8)	Input: *t* = 0.507, cap: *t* = 2.520; df = 6	6
	Input: WT:1.248 ± 0.335, 4Eki: 1.440 ± 0.583, cap: WT: 1.003 ± 0.142, 4Eki: 0.325 ± 0.075	Input: *p* = 0.784, cap: *p* = 0.005	eIF4A1		Input: *t* = 0.285, cap: *t* = 4.196; df = 6	
	1.088 ± 0.08499, 0.9525 ± 0.05977, cap: WT: 0.997 ± 0.222, 4Eki: 0.9175 ± 0.199	Input: *p* = 0.241, cap: *p* = 0.798	eIF4E		Input: *t* = 1.299 , cap: *t* = 0.267; df = 6	
	Input: WT:1.145 ± 0.078, 4Eki: 0.905 ± 0.099 , cap: WT: 1.002 ± 0.149, 4Eki: 0.830 ± 0.218	Input: *p* = 0.106, cap: *p* = 0.544	eIF4G		Input: *t* = 1.894, cap: *t* = 0.642; df = 6	
	Input: WT:1.025 ± 0.108, 4Eki: 0.98 ± 0.108 , cap: WT: 1.002 ± 0.185, 4Eki:0.10.2 ± 0.185	Input: *p* = 0.839, cap: *p* = 0.382	Eprs		Input: *t* = 0.206, cap: *t* = 1.063; df = 14	
	Input: WT: 0.998 ± 0.091, 4Eki: 0.901 ± 0.091 , cap: WT: 0.998 ± 0.254, 4Eki: 0.775 ± 0.254	Input: *p* = 0.106, cap: *p* = 0.544	Gapdh		Input: *t* = 1.060, cap: *t* = 0.880; df = 14	

## Results

### Loss of eIF4E phosphorylation does not affect hippocampal learning, memory, or L-LTP

**Figure 1.**
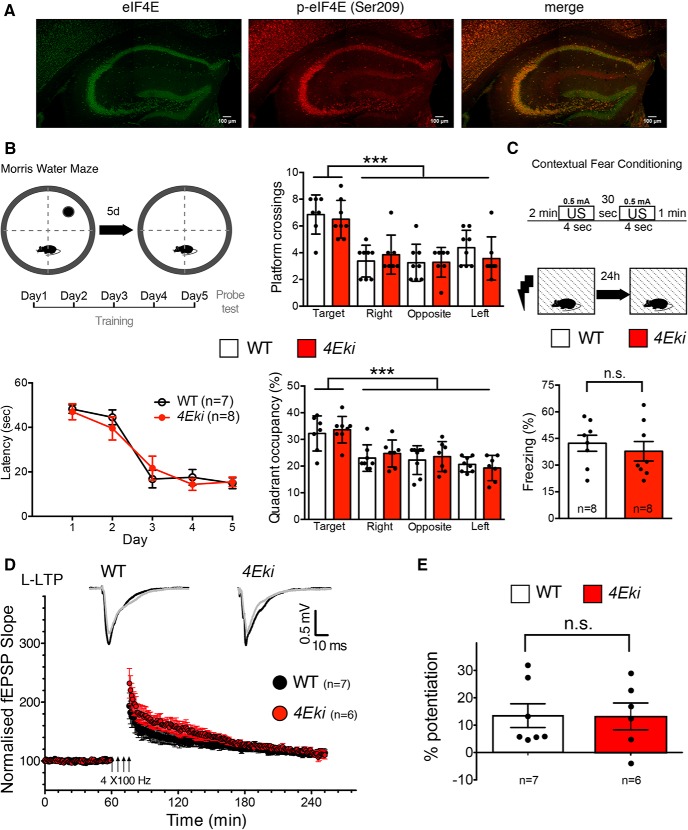
Intact spatial learning and memory, contextual fear memory, and L-LTP in *4Eki* mice. ***A***, Representative confocal images of immunofluorescent staining of WT dorsal hippocampi with antibodies against total and phospho-Ser209 eIF4E. Scale bar, 100 μm. ***B***, MWM task. Left, Graphic depiction of experimental design; latency (seconds) to find hidden platform during experimental days. Right, Platform crossings and quadrant occupancy during probe test (WT, *n* = 7; *4Eki*, *n* = 8). ***p < 0.001 (repeated-measures ANOVA with Tukey's *post hoc*). ***C***, CFC in *4Eki* mice. Percentage freezing 24 h after initial shock (WT, *n* = 8; *4Eki*, *n* = 8). ***D***, CA1 L-LTP recordings in *4Eki* mice. Normalized fEPSP slope over time (min) for 240 min. ***E***, Summary quantification of percentage potentiation for L-LTP. (Student's *t* test).

eIF4E is highly expressed throughout the hippocampal formation (Fig. 1*A*). To examine the role of eIF4E Ser209 phosphorylation in the hippocampus, we subjected WT and *Eif4e^Ser209Ala^* phospho-mutant knock-in mice (*4Eki*) (Gkogkas et al., 2014) to hippocampus-dependent behavioral tests. First, we examined spatial memory in the MWM (Fig. 1*B*). *4Eki* mice were indistinguishable from WT mice during the learning phase, as they displayed comparable latency to find the hidden platform, and comparable numbers of platform crossings (Fig. 1*B*). Quadrant occupancy during the probe test on day 6 was not different between WT and *4Eki* mice (Fig. 1*B*). Second, we assessed long-term contextual fear memory using a CFC task (Fig. 1*C*). In line with MWM data, contextual memory was intact in *4Eki* mice, as the percentage of freezing in response to context was not different from WT mice (Fig. 1*C*). These data indicate that hippocampus-dependent contextual memory is not affected by the lack of eIF4E phosphorylation.

We next measured LTP in CA1 hippocampal area, a form of plasticity that is MAPK- and protein synthesis-dependent (Frey et al., 1988; English and Sweatt, 1997). Four trains of high-frequency stimulation (4HFS) of the Schaffer collateral-CA1 synapses elicited long-lasting potentiation of fEPSPs in WT slices (Fig. 1*D*). The 4HFS-induced potentiation was not different in slices prepared from *4Eki* mice compared with WT (Fig. 1*D*,*E*). Together, mutating the single phosphorylation site on eIF4E (which lies downstream of the MAPK/ERK/MNK pathway and upstream of translation initiation) does not impair hippocampus-dependent learning and memory, or CA1 hippocampal L-LTP.

### Phospho-eIF4E regulates the translation of a subset of mRNAs

**Figure 2.**
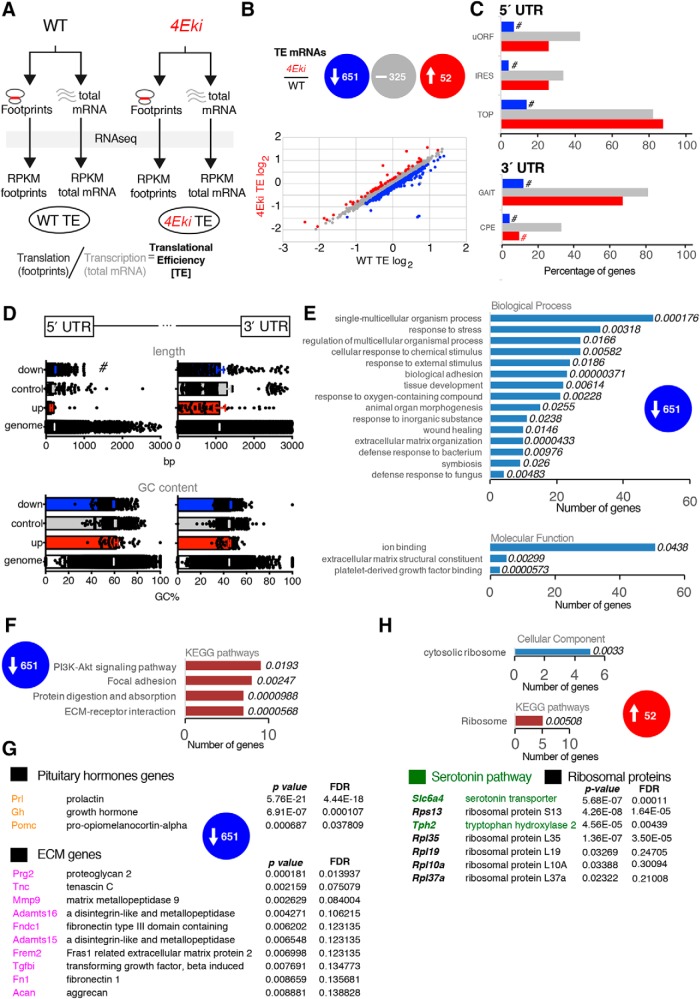
Ribosome profiling reveals preferential translation of a subset of mRNAs in the forebrain of *4Eki* mice. ***A***, Experimental design to assess genome-wide translational efficiency of mRNAs using ribosome profiling in whole brain tissue from WT and *4Eki* mice. ***B***, log_2_ TE Plot showing translationally upregulated (red), downregulated (blue), and control (gray) mRNAs in *4Eki* versus WT libraries (*p* < 0.05 and 0.75 ≥ TE ratio ≤ 1.5). Gray represents unchanged mRNAs; *n* = 2 for footprints and mRNA. ***C***, UTR analysis using RegRNA in downregulated (651; blue), upregulated (52; red), and control (325; gray) mRNAs in *4Eki*, compared with WT. Percentage of genes containing one or more of the depicted RNA sequence elements in 5′- or 3′-UTR. ^#^Categories in downregulated or upregulated mRNAs, which are underrepresented compared with control mRNAs. (Fig. 2-1***A***, Fig. 2-1***B***, Fig. 2-1***C*,*D***). ***D***, Length and GC content analysis of differentially translated mRNAs. Length (bp) or percentage of GC content is displayed for 5′-UTR (left) or 3′-UTR (right). ^#^*p* < 0.05 difference from all other categories (one-way ANOVA with Tukey's *post hoc*); all other multiple comparisons between groups are not significant. ***E***, Gene ontology analysis of 651 downregulated genes; plots for biological process, molecular function, and cellular component with number of genes in each category. *p* values next to each category are shown. ***F***, KEGG pathway analysis for downregulated genes. ***G***, Major genes downregulated in ribosome profiling organized in two categories: pituitary hormone genes and ECM genes with *p* value and FDR. ***H***, Gene ontology analysis of 52 upregulated genes; plots for cellular component with number of genes in each category and *p* values and KEGG pathway analysis. Major genes upregulated in ribosome profiling organized in two categories: serotonin and ribosomal proteins; *p* value and FDR are shown for downregulated and upregulated genes.

Given the unexpected result that eIF4E phosphorylation is not required for key forms of hippocampal memory formation and synaptic plasticity, we sought to elucidate the role of phospho-eIF4E in the brain by performing genome-wide analysis of mRNA translation, with the ribosome profiling methodology (Ingolia et al., 2012). Using forebrain tissue (including hippocampus) from WT and *4Eki* mice, we generated libraries for RNA sequencing from randomly fragmented total RNA (a proxy for transcription) and from ribosome-protected footprints following RNase digestion (a proxy for translation), to measure the translational efficiency of mRNAs (Fig. 2*A*). We did not observe a significant change in global translation or transcription in 4Eki forebrain (Fig. 2*B*), in accordance with previous reports (Gkogkas et al., 2014). The high quality of footprint and mRNA libraries is evidenced by the following: (1) the *r*^2^ of RPKM between replicates, which is >0.99 for both footprints and total mRNA (Fig. 2-1*A*); (2) the canonical distribution of footprint size (28–32 nt) and of read distribution within the 3 frames (Fig. 2-1*B*); and (3) principal components and clustering analysis of replicates (Fig. 2-1*C*,*D*). We found that, even though the Ser209Ala mutation does not affect global translation, it regulates the translational efficiency of a subset of mRNAs (Fig. 2*B*). The translation of 651 mRNAs was significantly downregulated (*4Eki*/WT ratio ≤0.75, *p* < 0.05), whereas the translation of 52 mRNAs was significantly upregulated (*4Eki*/WT ratio ≥1.5, *p* < 0.05) (Fig. 2*B*).

10.1523/JNEUROSCI.2673-17.2018.f2-1 Figure 2-1 **Reproducibility and quality of RPF data. A.** Reproducibility plots for WT and 4Eki sequenced libraries (for replicates of total mRNA and footprints (grey corresponds to data points with <128 reads). **B.** Frequency and length of mapped reads and fraction of reads within start codon window for the 3 frames for total mRNA and footprint libraries. Principle components analysis (PCA) and clustering analysis dendrogram for **C.** Translation and **D.** Transcription for the two biological replicates used for WT (WT1, WT2) or 4Eki (KI1, KI2). Download Figure 2-1, TIF file

Because UTRs harbor sequence elements, which may explain changes in translational efficiency, we analyzed *4Eki*-sensitive mRNA 5′- and 3′-UTRs, along with 325 mRNAs (control group) that were not regulated by phospho-eIF4E in our ribosome profiling experiment (Fig. 2*C*), using the RegRNA suite (Huang et al., 2006). The 5′-UTRs of downregulated, but not upregulated, mRNAs contain a reduced number of upstream open reading frames (uORFs), internal ribosome entry sites (IRESs), and terminal oligopyrimidine tract (TOP) compared with the control group (Fig. 2*C*). The 3′-UTRs of downregulated mRNAs, but not of upregulated, harbor a significantly reduced number of GAIT elements, compared with the control and upregulated mRNA groups (Fig. 2*C*). The incidence of cytoplasmic polyadenylation elements (CPEs), both in downregulated and upregulated mRNAs, is reduced compared with the control group (Fig. 2*C*). These data suggest that the incidence of 5′ uORF, IRES, and 3′ GAIT elements in the UTRs of *4Eki*-downregulated mRNAs, compared with upregulated and control groups, may reveal a previously unidentified phospho-eIF4E-dependent translational control mechanism in the brain. Notably, we analyzed the length and guanine-cytosine content (GC%) in UTRs and detected a significant increase in the length of 5′-UTRs in downregulated mRNAs, compared with other mRNA groups, but not for GC% (Fig. 2*D*). 3′-UTR length or GC% was not different between gene groups (Fig. 2*D*).

To further understand the translational control mechanisms downstream of phospho-eIF4E in the brain, we performed gene ontology analysis for the downregulated (Fig. 2*E–G*) and upregulated genes (Fig. 2*H*). For the significantly downregulated genes group, we identified several biological process, molecular function, and cellular component categories (*p* < 0.05; Fig. 2*E*,*F*). Some key categories include response to stress, extracellular organization and ECM, biological adhesion, and defense response, whereas some key pathways were also identified (e.g., PI3K-Akt signaling pathway and ECM-receptor interaction) (Fig. 2*E*,*F*). Some of the major gene groups that are downregulated in *4Eki* forebrain are genes encoding for pituitary hormones and ECM genes (Fig. 2*G*), including *Mmp9*, which we have previously shown to be crucial for reversing behavioral, anatomical, and biochemical deficits in *Fmr1*^−/*y*^ mice (Gkogkas et al., 2014; Gantois et al., 2017). Conversely, in the upregulated genes group, the most enriched gene ontology category and pathway is the ribosome, whereas two major gene groups that are upregulated translationally include genes in the serotonin pathway and ribosomal protein coding genes (Fig. 2*H*). Together, these data suggest that, downstream of MAPK/ERK, eIF4E phosphorylation does not affect global translation but preferentially regulates the synthesis of certain proteins by modulating their mRNA translation via 5′- and 3′-UTR elements, such as GAIT. Importantly, the list of regulated mRNAs points toward a role of phospho-eIF4E in ECM regulation, pituitary hormones, the serotonin pathway, and ribosomal proteins.

### Exaggerated inflammatory response and reduced serotonin levels in *4Eki* brain

**Figure 3.**
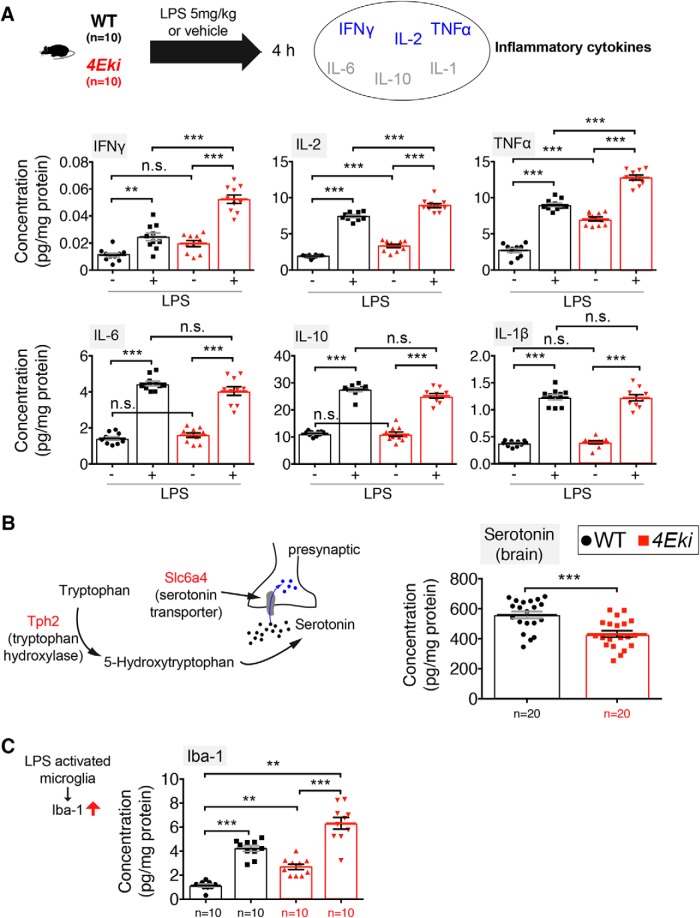
Exaggerated inflammatory responses and reduced serotonin levels in *4Eki* brain. ***A***, Quantitative ELISA for 6 mouse inflammatory cytokines in WT and *4Eki* forebrains (*n* = 10 for each genotype). Blue represents Th1 cytokines. Gray represents Th2 cytokines. ***B***, Left, Serotonin pathway genes upregulated in *4Eki* brain (red). Quantitative ELISA for serotonin (5-HT) in WT and *4Eki* forebrains (*n* = 20 for each genotype). Normalized concentration (pg/mg) is shown for all experiments. ***C***, Quantitative ELISA for Iba-1, a marker of activated microglia (*n* = 10 for each genotype). ***A***, ***C***, ***p < 0.001 (one-way ANOVA with Bonferroni's *post hoc*). **p < 0.01 (one-way ANOVA with Bonferroni's *post hoc*). ***B***, ***p < 0.001 (Student's *t* test).

To further investigate the role of phospho-eIF4E in the brain, we proceeded to identify potential phenotypic changes, which could result from the aberrant translation of specific categories of mRNAs in *4Eki* brain (Fig. 2). We hypothesized that inflammatory responses may be altered in *4Eki* mice, given the known link of phospho-eIF4E and eIF4E to innate immunity (Colina et al., 2008; Herdy et al., 2012) and because many inflammatory mRNAs harbor GAIT elements in their 3′-UTRs (Mukhopadhyay et al., 2009), similarly to our upregulated mRNAs (Fig. 2*C*). The mRNA 3′-UTR GAIT element is a “gatekeeper” of inflammatory gene expression (Mukhopadhyay et al., 2009). Therefore, we set out to measure inflammatory reponses in forebrain lysates using quantitative ELISA for 6 major cytokines. Treatment of *4Eki* mice with LPS (strain O111:B4, 5 mg/kg, intraperitoneally) led to a significantly higher expression of distinct cytokines 4 h after injection in *4Eki* mouse forebrain, compared with WT (Fig. 3*A*). In *4Eki* brain, we detected a significant increase in IL-2 and TNFα expression, both at baseline and following LPS stimulation, compared with WT (Fig. 3*A*). For IFNγ, we detected a significant upregulation in *4Eki* versus WT only following LPS stimulation, but not at baseline (Fig. 3*A*), whereas for IL-6, IL-10, and IL-1B there were no differences between *4Eki* and WT mice (Fig. 3*A*). Interestingly, IL-2, TNFα, and IFNγ are produced by Th1-type T-cell subsets, whereas IL-6, IL-10, and IL-1B by Th2-type (Romagnani, 2000).

We further reasoned that the translational upregulation of the serotonin uptake receptor (Slc6a4) and the enzyme tryptophan hydroxylase (Tph2) (Fig. 2*H*) would be accompanied by changes in the amount of serotonin in the 4Eki brain, as previously shown (Charoenphandhu et al., 2011; Zhang et al., 2012; Yohn et al., 2017). Using quantitative ELISA, we measured a decrease in tissue levels of serotonin in 4Eki forebrain, compared with WT (Fig. 3*B*). Furthermore, we also detected an increase in Iba-1 at baseline and following LPS stimulation in *4Eki* mice, compared with WT (Fig. 3*C*), suggesting that microglia are activated in the Ser209Ala mouse model.

Together, these data suggest that the elaborate translational landscape downstream of phospho-eIF4E elicits complex alterations in the brain consisting of changes in inflammatory responses and serotonergic function.

### Depression and anxiety-like behaviors in *4Eki* mice

**Figure 4.**
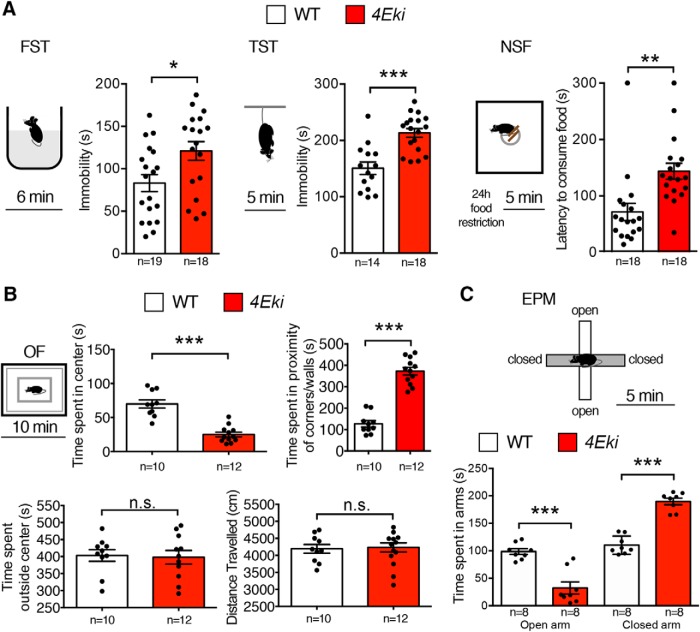
Depression and anxiety-like behaviors in *4Eki* mice. ***A***, Immobility time (seconds) as an indicator of depression-like behaviors in FST (left) and TST (middle) in WT(n = 14) and *4Eki* (n = 18) mice. NSF (right), latency to start feeding in a novel environment, as a proxy for depression/anxiety-mediated hypophagia in WT and *4Eki* (*n* = 18 each) mice. ***B***, Open field exploration test (OF) in WT (*n* = 10) and *4Eki* (*n* = 12) mice, as a measure of anxiety. Time (seconds) spent in the center square, in proximity of corners or walls, or outside the center square and total distance traveled. ***A***, ***B***, *p < 0.05 (Student's *t* test). **p < 0.01 (Student's *t* test). ***p < 0.001 (Student's *t* test). ***C***, EPM in WT (*n* = 8) and *4Eki* (*n* = 8) mice, as a measure of anxiety. Time (seconds) spent in the open or closed arms of the elevated maze. ***C***, ***p < 0.001 (one-way ANOVA with Bonferroni's *post hoc*).

There is a strong link between serotonin, pituitary hormones such as prolactin, and depression/anxiety (Bob et al., 2007; Yohn et al., 2017). Moreover, phospho-eIF4E is upregulated in response to chronic treatment with the SSRI antidepressant fluoxetine (Dagestad et al., 2006). Thus, we reasoned that the pathways regulated by eIF4E phosphorylation could be linked to depression. To test this hypothesis, we subjected WT and *4Eki* mice to the FST and TST, which have been shown to model depression-like behaviors in mice by assessing passive immobility after a few minutes of futile struggling (Cryan and Holmes, 2005). *4Eki* mice remained immobile longer than WT mice in both FST and TST tests, suggesting a depression-like phenotype (Fig. 4*A*). To further study the depression-like phenotype of the *4Eki* mice, we used the NSF, which measures the latency of a mouse to start feeding in a novel environment, following 24 h food restriction. It has been extensively shown that mouse models of depression display increased latencies to initiate feeding in the NSF test (hyponeophagia) (Dulawa and Hen, 2005), whereas chronic antidepressants were shown to reduce this latency (Britton and Britton, 1981). *4Eki* mice required a significantly higher amount of time per session to initiate feeding in NSF, compared with WT (Fig. 4*A*). Furthermore, we examined *4Eki* mice for anxiety-like behaviors using the open field test (Fig. 4*B*). *4Eki* mice spent significantly less time in the central region of the arena and significantly more time in proximity to walls or corners, suggesting elevated anxiety; however, the time spent outside the central square and total distance traveled were similar between *4Eki* and WT mice, indicating that locomotion was not affected (Fig. 4*B*). In line with these findings, we detected an anxiety-like phenotype in *4Eki* mice subjected to the EPM (Fig. 4*C*). *4Eki* mice, compared with WT, spend significantly less time in the open and significantly more time in the closed arms of the maze (Fig. 4*C*). In summary, these data indicate that *4Eki* mice display anxiety and depression-like behaviors.

### Chronic fluoxetine treatment does not rescue depression-like behaviors in *4Eki* mice

**Figure 5.**
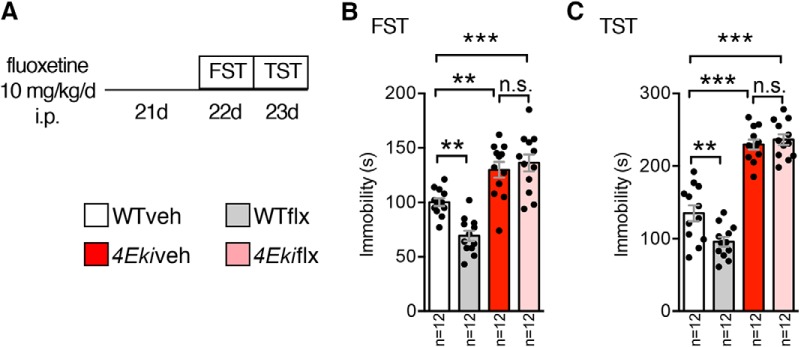
Chronic fluoxetine intraperitoneal treatment does not reverse depression-like behaviors in *4Eki* mice. ***A***, Outline of chronic fluoxetine regimen. Intraperitoneal injection of 10 mg/kg/d for 21 d reduces immobility time (seconds) in WT (*n* = 12) but not *4Eki* (*n* = 12) mice. ***B***, FST. ***C***, TST. **p < 0.01, ***p < 0.001 (one-way ANOVA with Bonferroni's post-hoc).

Chronic fluoxetine treatment induced phosphorylation of eIF4E at Ser209 (Dagestad et al., 2006) and alleviated depression-like phenotypes in mice (Dulawa et al., 2004). Thus, we hypothesized that the chronic antidepressant effect of fluoxetine is mediated via stimulation of eIF4E phosphorylation (Fig. 5*A*). Chronic (21 d) intraperitoneal treatment of WT mice with fluoxetine (10 mg/kg/d) led to an ∼25% decrease in immobility in both FST and TST tests (Fig. 5*A–C*), which is in accordance with previous reports (Dulawa et al., 2004). Strikingly, in *4Eki* mice, fluoxetine did not affect immobility in either test (Fig. 5*B*,*C*), indicating that phospho-eIF4E is required for the antidepressant action of fluoxetine.

### Reduced cap binding of rpL13a and eIF4A1 in *4Eki* mice

**Figure 6.**
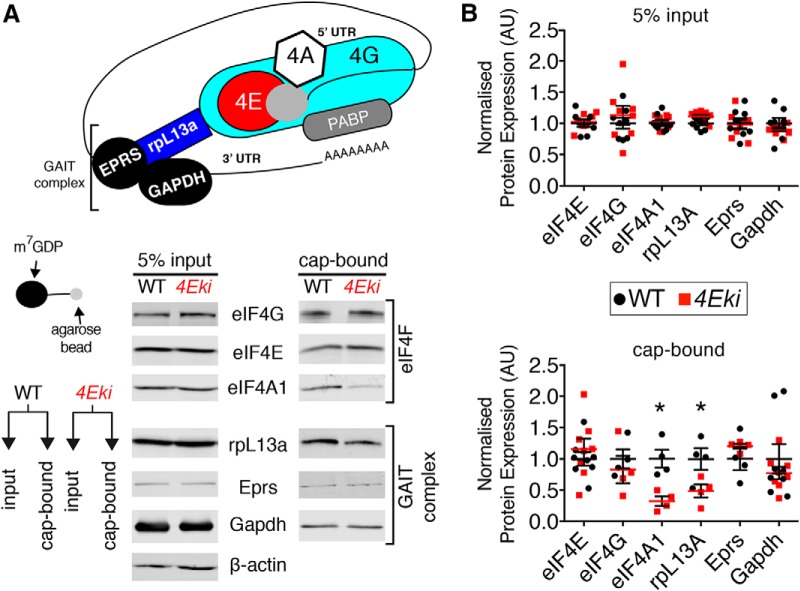
Altered cap binding of GAIT complex protein rpL13a and eIF4A1 in *4Eki* brains. ***A***, Cap-column (m^7^GDP) pulldown from forebrain lysates (WT and *4Eki*; *n* = 4 per genotype or *n* = 8 for Eprs, Gapdh). Left, Diagram of the closed loop model of translation depicting binding of repressive 3′-UTR GAIT elements to 5′-UTR cap-bound eIF4F complex, via rpL13a and below a depiction of a cap-column agarose bead. Representative immunoblot images from cap-bound and input lysates probed with antibodies against the indicated proteins (eIF4E, eIF4G, eIF4A1 rpL13a, Eprs, and Gapdh; β-actin is the loading control). ***B***, Quantification of protein expression from input (5%) and cap-bound lysates. Protein expression (arbitrary units) normalized to input protein expression for cap-bound lysates and to β-actin for input lysates. *p < 0.05 (Student's *t* test).

UTR analysis of differentially translated mRNAs in the forebrain of *4Eki* mice revealed that upregulated mRNAs display a higher incidence of 3′-UTR GAIT elements, compared with downregulated mRNAs (Fig. 2*C*). GAIT elements repress translation by recruiting a complex of proteins (GAIT complex: rpL13a, Eprs and Gapdh) on mRNA 3′-UTR (Mukhopadhyay et al., 2009). Subsequently, the GAIT complex is bridged to the 5′-UTR cap-bound eIF4F, via direct interaction of the GAIT complex protein rpL13a and eIF4G (Fig. 6*A*). Reduced binding of GAIT complexes to eIF4F, when phospho-eIF4E is depleted, could explain the upregulation of a small subset of mRNAs containing 3′-UTR GAIT elements (*n* = 52; Fig. 2*C*,*H*) via translational disinhibition. Likewise, mRNAs with low incidence of 3′-UTR GAIT elements should not be affected to the same extent by this regulatory mechanism (Fig. 2*C*,*G*). To test this hypothesis, we performed cap-column pulldown of forebrain lysates using m^7^GDP agarose beads, followed by immunoblotting of cap-bound and of whole lysates as a control (Fig. 6*A*). By probing for key eIF4F proteins (eIF4E, eIF4G, and eIF4A), we can detect changes in their binding to the mRNA cap. By probing for the GAIT complex proteins rpL13a, Eprs, and Gapdh in cap-bound fractions, we can assess changes in GAIT complex-eIF4F binding; importantly, rpL13a bridges GAIT to eIF4F (Fig. 6*A*). We detected in *4Eki* forebrain lysates, decreased cap binding of rpL13a and of the eIF4F helicase eIF4A1, whereas eIF4E, eIF4G, Eprs, and Gapdh cap binding was not altered (Fig. 6*A*,*B*). Eprs and Gapdh cap binding was not altered in *4Eki* mice, which could be due to the fact that these proteins may interact with eIF4F as monomers, outside of the GAIT complex (Sampath et al., 2004). This is not the case for ribosomal protein rpL13a, as its main extraribosomal function is to bridge GAIT to eIF4F and mediate translational repression (Kapasi et al., 2007).

**Figure 7.**
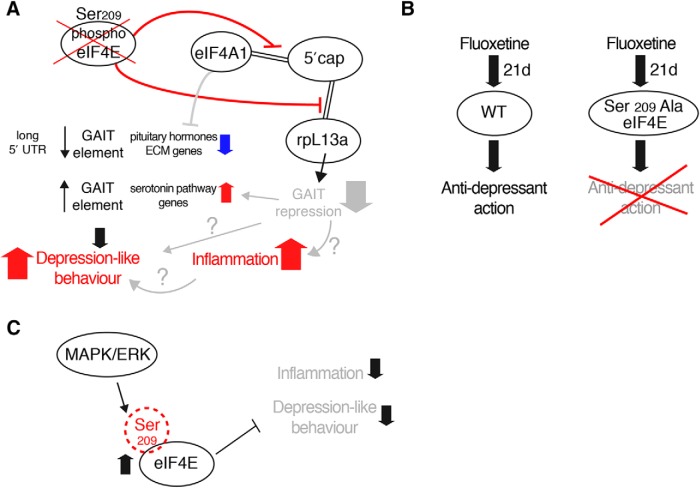
Depletion of eIF4E phosphorylation engenders inflammatory and depression-like phenotypes via selective translational control of a subset of mRNAs. ***A***, Ablation of the single phosphorylation site on eIF4E (Ser209→Ala) does not affect global protein synthesis, but rather the translation of a subset of mRNAs harboring GAIT elements, which engenders a depression-like phenotype in *4Eki* mice. *4Eki* mice also display increased expression of inflammatory cytokines, which could be linked to disinhibition of GAIT translational repression and possibly to depression-like phenotypes. Altered cap binding of the helicase eIF4A1 and/or of the GAIT complex protein rpL13a could be the mechanism underlying altered translation initiation following depletion of Ser209 eIF4E phosphorylation. ***B***, The SSRI fluoxetine requires eIF4E phosphorylation to exert its antidepressant action. ***C***, Phosphorylation of eIF4E promotes anti-inflammatory and antidepressant pathways.

Thus, ablation of the single phosphorylation site on eIF4E engenders selective translation of a subset of mRNAs, conceivably through altered cap binding and translation initiation mediated by mRNA UTR elements, such as GAIT (Fig. 7*A*).

## Discussion

We show that phospho-eIF4E plays a previously unidentified role in the brain, whereby its depletion engenders depression-like behaviors (Fig. 4) and resistance to the chronic antidepressant action of the SSRI fluoxetine (Fig. 7*B*). We also show that eIF4E phosphorylation is not required for major forms of hippocampal learning and memory and L-LTP (Fig. 1). We further demonstrate that a potential underlying mechanism involves the selective mRNA translation of GAIT element-containing mRNAs and of mRNAs harboring long 5′-UTRs (Fig. 7*A*). This multifaceted translational control pathway in 4Eki mouse brain may be responsible for the observed changes in inflammatory responses, serotonin levels, pituitary hormones, and the ECM (Figs. 2, 3), which could underlie the depression-like behaviors (Fig. 4) and the resistance to the antidepressant action of fluoxetine (Fig. 5).

Translational control by the MAPK pathway was shown to be crucial for hippocampal synaptic plasticity, learning, and memory (Kelleher et al., 2004). Contrary to the prediction that ablation of phospho-Ser209 in eIF4E would recapitulate MAPK deletion phenotypes, we found that in Ser209Ala mutant mice (*4Eki*), hippocampal learning and memory, as well as a major form of long-term synaptic plasticity (L-LTP) are intact (Fig. 1). It is generally believed that L-LTP and long-term memory require new protein synthesis (Frey et al., 1988). We show, for the first time, that the phosphorylation of eIF4E downstream of MAPK/ERK is not required for L-LTP (Fig. 1*D*). We cannot rule out the possibility that phospho-eIF4E is essential for other forms of synaptic plasticity (Panja et al., 2014) or that it is important in brain regions outside the hippocampus. We also cannot exclude the presence of compensatory mechanisms in *4Eki* mice (e.g., mTORC1 activation), which could substitute for the loss of eIF4E phosphorylation. Alternatively, MAPK/ERK may regulate hippocampal synaptic plasticity, learning, and memory by phosphorylating other translation initiation factors.

Ribosome profiling in the brain of 4Eki mice revealed translational downregulation of several mRNAs (encoding for ECM genes and pituitary hormones) (Fig. 2*G*). eIF4E phosphorylation was previously suggested to control cancer metastasis (Furic et al., 2010; Robichaud et al., 2015), by controlling ECM function and in particular the translation of MMPs, such as MMP-9 (Furic et al., 2010; Gkogkas et al., 2014; Gantois et al., 2017). Thus, it will be important to further investigate the role of ECM regulation downstream of phospho-eIF4E in the brain. Control of pituitary mRNA translation is a novel function assigned to phospho-eIF4E; and apart from its link to depression, it will be important to examine its potential links to other neuropsychiatric or neurodevelopmental disorders or cancer (e.g., pituitary adenomas). On the other hand, serotonin pathway and ribosomal protein coding genes are upregulated in *4Eki* brain (Fig. 2*H*). Given the interplay between the hypothalamic-pituitary-adrenal axis, serotonin and dopamine (Hamon and Blier, 2013; Hoogendoorn et al., 2017), we are proposing a new translational control pathway (via phospho-eIF4E) implicated in this regulation, which may be modulated pharmacologically. The ribosome profiling strategy was invaluable in identifying phospho-eIF4E-regulated transcripts, and subsequently phenotypic changes. However, it did not reveal cell-type-specific alterations in translation, which could further elucidate the mechanisms underlying the depression-like phenotypes observed in *4Eki* mice. Given that we detected inflammatory changes in Iba-1 (a marker of microglia activation; Fig. 3*C*), it would be imperative to perform cell-type-specific profiling of translation in neuronal and non-neuronal cells (e.g., microglia) using methodologies, such as TRAP (Heiman et al., 2014). Nevertheless, our translational profiling revealed that ablation of eIF4E phosphorylation downregulates the translation of a large subset of mRNAs, without affecting global translation, and upregulates the translation of a very small subset of mRNAs (Fig. 2). Overall, these data suggest that eIF4E phosphorylation promotes translation initiation.

Proinflammation programs in 4Eki mice (Figs. 2, 3) could be causal for the depression-like behaviors observed in these mice (Fig. 4). Depression is frequently comorbid with many inflammatory illnesses (Liu et al., 2017), while antidepressants can decrease inflammatory responses (Wiedlocha et al., 2018), suggesting that depression and inflammation are closely linked. Conceivably, proinflammatory responses in *4Eki* brain could be linked to depression-like behaviors: (1) by GAIT mRNA translational disinhibition (Figs. 2, 6), linked to inflammation (Fig. 3); (2) through the known link of eIF4E and enhanced Type I IFN production (Colina et al., 2008); or (3) as a result of enhanced activity of NF-κB following translational downregulation of its inhibitor IκBα in *4Eki* (Herdy et al., 2012). Indeed, we observed a baseline and LPS-stimulated upregulation of Th1-type (Romagnani, 2000) cytokines: IFNγ, TNFα, and IL-2 (Fig. 3*A*). Notably, a shift in the Th1/Th2 balance in favor of Th1 cytokine expression cytokines was shown to be linked to depression (Gabbay et al., 2009; Maes, 2011) and other neuropsychiatric disorders (Hickie and Lloyd, 1995).

We identified exacerbated immobility/“despair-like,” hyponeophagy and anxiety-like behaviors in *4Eki* mice, which are reminiscent of human depression/anxiety (Fig. 4). The link between phospho-eIF4E and depression is further strengthened by the fact that chronic fluoxetine treatment (10 mg/kg for 21 d) requires eIF4E phosphorylation to exert its antidepressant effect (Fig. 5). Fluoxetine also recruits other pathways upstream of the translation initiation machinery, such as mTORC1 (Liu et al., 2015). While the connection between inflammation and depression is still under investigation, our data highlight a new translational control pathway, which may underlie the chronic antidepressant action of SSRIs and could be exploited to design novel antidepressants by boosting eIF4E phosphorylation (Fig. 7*C*).

In addition, we have further elucidated the mechanism of translational control downstream of phospho-eIF4E by identifying key 5′- and 3′-UTR sequence elements, and changes in signaling, which may confer specificity to phospho-eIF4E translational control (Fig. 2*C*). From the UTR analysis, we identified an underrepresentation of uORF, IRES, TOP, CPE, and GAIT elements in *4Eki*-downregulated mRNAs. CPE elements may regulate translation of *4Eki*-sensitive mRNAs, which could be explained through changes in the activity of poly(A)-binding protein, which prompts mRNA circularization by bridging 5′ eIF4G to 3′ poly(A) tail (Smith et al., 2014). Furthermore, translation of uORF-containing mRNAs is regulated by the eIF2α pathway in the brain (Costa-Mattioli et al., 2007). Even though we did not detect any changes in eIF2α phosphorylation in *4Eki* mice (data not shown), mTORC1 may regulate uORF-containing mRNA translation (Schepetilnikov et al., 2013). Likewise, we did not detect significant changes in IRES translation in *4Eki* mice (data not shown). The translation of TOP mRNAs (e.g., ribosomal protein coding mRNAs) was previously shown to be mTORC1-sensitive (Avni et al., 1997; Thoreen et al., 2012), which is in line with our GO analysis (Fig. 2*G*,*H*; KEGG pathways).

The presence of GAIT sequence elements in the 3′-UTR of proinflammatory mRNAs suppresses their translation (Mukhopadhyay et al., 2008). A key event in this process is the binding of rpL13a, a core constituent of the GAIT protein complex, to the 5′ cap by direct binding to eIF4G (Fox, 2015). Genetic depletion of eIF4E Ser209 phosphorylation leads to reduced binding of rpL13a to the 5′ cap (Fig. 6), predicting that in *4Eki* brain there would be translational disinhibition of mRNAs harboring GAIT elements. Indeed, *4Eki*-downregulated mRNAs have a low incidence of 3′-UTR GAIT elements, which could explain why they are not affected by phospho-eIF4E-mediated, GAIT complex-dependent translational disinhibition. This also suggests that downregulation of the 651 mRNAs probably occurs via a different mechanism. Conversely, upregulated mRNAs display a significantly higher incidence of 3′-UTR GAIT elements (Fig. 2*C*). Concomitantly, *4Eki* brains exhibit exaggerated expression of proinflammatory cytokines, which could be explained by GAIT complex-mediated disinhibition of mRNAs coding for cytokines (Fig. 3*A*). Furthermore, cap-pulldown of the helicase eIF4A1 is significantly reduced in *4Eki* forebrain (Fig. 6) and is accompanied by increased length of 5′-UTRs in *4Eki*-downregulated mRNAs, compared with other groups (Fig. 2*D*). Thus, it is possible that phospho-eIF4E requires the helicase eIF4A1 to resolve long 5′-UTRs, which is in accordance with previous reports linking eIF4E to eIF4A1 activity (Feoktistova et al., 2013). This mechanism could explain the translational downregulation of the 651 mRNAs. Thus, our ribosome profiling data along with the biochemical investigation of cap complex formation in the brains of *4Eki* mice have revealed potential mechanisms for the observed selective translational control. However, further work is required to build a comprehensive model for the synergistic action of UTR elements, such as GAIT, uORF, IRES, TOP, and CPE, downstream of eIF4E phosphorylation.

In conclusion, phospho-eIF4E-dependent translation of GAIT element-containing mRNAs may constitute a unifying mechanistic explanation as to how dysregulated translational control of specific mRNAs could be causal for inflammation and depression, without affecting general translation.
