# Supplementary figures and images for: Loss of eIF4E Phosphorylation Engenders Depression-like Behaviors via Selective mRNA Translation
Source: J Neurosci. 2018 Feb 21;38(8):2118–33. doi: 10.1523/JNEUROSCI.2673-17.2018 (PMC5824745; doi:10.1523/JNEUROSCI.2673-17.2018)

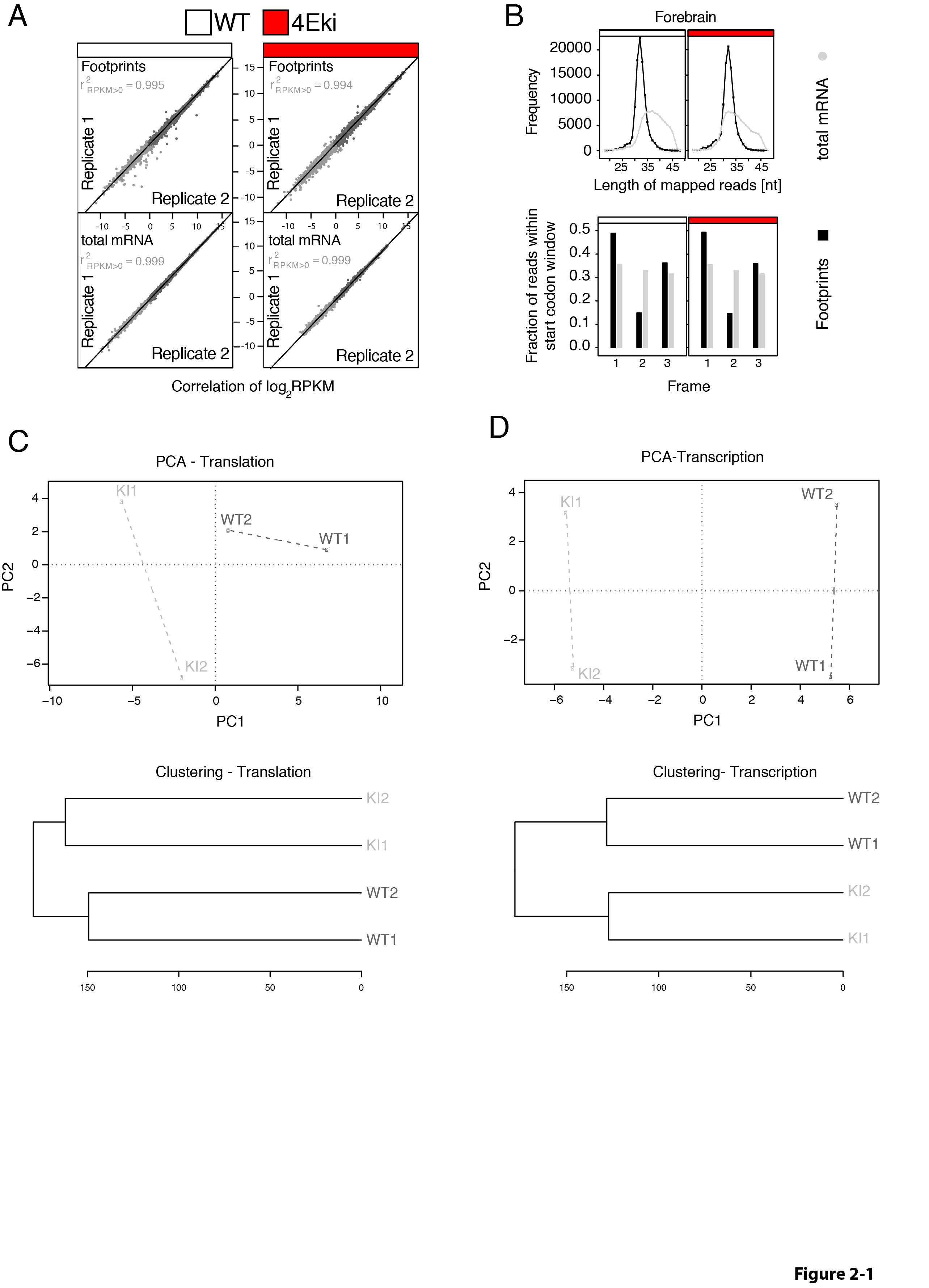

Supplement: Figure 2-1 [file zns999180550so2.tif]
